# Clinical impact of the two ART resistance markers, K13 gene mutations and DPC3 in Vietnam

**DOI:** 10.1371/journal.pone.0214667

**Published:** 2019-04-02

**Authors:** Maria Carmina Pau, Antonella Pantaleo, Ioannis Tsamesidis, Ha Hoang, Anh Tuan Tran, Thi Lien Hanh Nguyen, Thi Hang Giang Phan, Phuong Anh Ton Nu, Thi Minh Chau Ngo, Giuseppe Marchetti, Evelin Schwarzer, Pier Luigi Fiori, Philip S. Low, Chien Dinh Huynh, Francesco Michelangelo Turrini

**Affiliations:** 1 Department of Biomedical Sciences, University of Sassari, Sassari, Italy; 2 Duy Tan University, Danang, Vietnam; 3 Huong Hoa District Health Center, Quang Tri, Vietnam; 4 Quy Nhon Institute of Malaria, Parasitology and Entomology, Quy Nhon, Vietnam; 5 Department of Immunology and Pathophysiology, Hue University, Hue City, Vietnam; 6 Department of Parasitology, Hue University, Hue City, Vietnam; 7 Department of Oncology, University of Turin, Turin, Italy; 8 Purdue Center for Drug Discovery and Department of Chemistry, Purdue University, West Lafayette, Indiana, United States of America; 9 VINMEC Heath Care System; Hai Ba Trung District, Hanoi, Vietnam; Institut national de la santé et de la recherche médicale - Institut Cochin, FRANCE

## Abstract

**Background:**

In Vietnam, a rapid decline of *P*. *falciparum* malaria cases has been documented in the past years, the number of *Plasmodium falciparum* malaria cases has rapidly decreased passing from 19.638 in 2012 to 4.073 cases in 2016. Concomitantly, the spread of artemisinin resistance markers is raising concern on the future efficacy of the ACTs. An evaluation of the clinical impact of the artemisinin resistance markers is therefore of interest.

**Methods:**

The clinical effectiveness of dihydroartemisinin-piperaquine therapy (DHA-PPQ) has been evaluated in three districts characterized by different rates of ART resistance markers: K13(C580Y) mutation and delayed parasite clearance on day 3 (DPC3). Patients were stratified in 3 groups a) no markers, b) one marker (suspected resistance), c) co-presence of both markers (confirmed resistance). In the studied areas, the clinical effectiveness of DHA-PPQ has been estimated as malaria recrudescence within 60 days.

**Results:**

The rate of K13(C580Y) ranged from 75.8% in Krong Pa to 1.2% in Huong Hoa district. DPC3 prevalence was higher in Krong Pa than in Huong Hoa (86.2% vs 39.3%). In the two districts, the prevalence of confirmed resistance was found in 69.0% and 1.2% of patients, respectively. In Thuan Bac district, we found intermediate prevalence of confirmed resistance. Treatment failure was not evidenced in any district. PPQ resistance was not evidenced. Confirmed resistance was associated to the persistence of parasites on day 28 and to 3.4-fold higher parasite density at diagnosis. The effectiveness of malaria control strategies was very high in the studied districts.

**Conclusion:**

No treatment failure has been observed in presence of high prevalence of ART resistance and in absence of PPQ resistance. K13(C580Y) was strongly associated to higher parasitemia at admission, on days 3 and 28. Slower parasite clearance was also observed in younger patients.

## Introduction

Artemisinin (ART) resistance was first described in Cambodia [1–4] and, successively, in different areas of the Greater Mekong sub-region (GMS) [5–9] including Vietnam[10,11]. ART resistance is defined as delayed parasite clearance (DPC) following artemisinin-based combination therapy (ACT) resulting in a prolonged parasite clearance time [12,13].

A molecular marker of ART resistance (mutations on P. *falciparum* K13 propeller gene) was found to be associated with DPC [8,11,14]. Some K13 mutations and DPC on day 3 (DPC3) are markers that are currently used to identify ART resistance, the co-presence of the two markers, is defined as “confirmed endemic artemisinin resistance” [12].

Anyway, the definition and the clinical impact of ART resistance are still controversial [15]. As a matter of facts, WHO updates from 2011 to 2018 reveal that the definition of ART resistance is undergoing a constant evolution. The majority of reports on artemisinin resistance did not take in consideration its effects on clinical treatment failure [8,11,16–20]. A minority of reports displayed that the presence of both ART resistance markers was associated to clinical failure [21–23] while others reports did not confirm this observation [24,25]. On the other hand, the co-occurrence of ART resistance and resistance to the partner drugs was described to be associated to high rates of clinical treatment failure [23,26]. In addition, the available ART resistance markers are affected by some technical limitations: i) their sensitivity and specificity are still undefined [27]; ii) resistance to mefloquine and piperaquine is, by itself, a demonstrated cause of clinical treatment failure of ACTs but the absence of a genetic marker cannot exclude drug resistance; iii) the rate of clearance of parasitized erythrocytes is multi-factorial being influenced by resistance to anti-malarial drugs and host factors, such as immunity and mutations affecting erythrocyte viability (G6PD deficiency and thalassemias) that may vary in the different areas.

In Vietnam high prevalence of the available ART resistance markers has been observed in some provinces [28,29] but the evidence of clinical treatment failure has been reported in one district with demonstrated resistance to piperaquine [23]. Although the national program for the eradication of malaria has provided very promising results, the increasing frequency of ART resistance markers [28] has raised concerns due to many previous episodes of resurgence that have been already observed worldwide following phases of apparent malaria control.

The objective of the present study is to evaluate the impact of different rates of ART resistance markers found in three Vietnamese districts on the efficacy of the current anti-malarial therapy. As standard index to assess ACT efficacy we utilized malaria recrudescence within 60 days from ACT treatment.

We also evaluated the interference of some known confounding factors such as mutations affecting erythrocytes and piperaquine resistance. The effectiveness of malaria control strategies has been estimated in the different areas (rate of decay of malaria prevalence and prevalence of asymptomatic carriers) to exclude a possible interference.

## Materials and methods

### Ethical clearance

The study was conducted in accordance with Good Clinical Practice guidelines and the Declaration of Helsinki and was approved by the Hue University of Medicine and Pharmacy Ethical Board. Written informed consent was obtained from the participants or from the legal representative of children aged less than 18 years before entering the study.

### Study sites, participants and sample collection (Clinical procedures)

This study was conducted from 2012 to 2016 in three areas of Vietnam: (i) the Huong Hoa district of Quang Tri province located in the North Central Coast region at the Laotian border; (ii) the Krong Pa district of Gia Lai province located in the Central Highlands) at the Cambodian border; and (iii) the Thuan Bac district of Ninh Thuan province located in the South Central Coast. In Huong Hoa, blood samples were collected from 84 malaria patients in 2014. In Krong Pa and Thuan Bac, blood samples were collected from 29 and 20 malaria patients, respectively, in 2015. Table 1 shows the major characteristics of the subjects enrolled in this study.

**Table 1:** Characteristics of subject registered in the studied areas.

Characteristics	Huong Hoa		Krong Pa		Thuan Bac
	General population	Malaria patients	General population	Malaria patients	Malaria patients
**Number of samples**	763	84	200	29	20
**Female**	41%	38%	57%	17.2%	60%
**Male**	59%	62%	43%	82.8%	40%
**Ethnicity (%)**	**Vân Kiều** (93.4%) **Pa Cô** (4.8%) **Kihn** (1.8%)	**Vân Kiều** (100%)	**Jarai** (97%) **Xa Deng** (3%)	**Jarai** (100%)	**Ra Glai** (100%)
**Age range N (%)**					
**≤5 yr**	63 (8.2%)	4 (4.8%)	8 (4.0%)	0 (0.0%)	0 (0.0%)
**6–10 yr**	110 (14.4%)	14 (16.7%)	27 (13.5%)	1 (3.4%)	2 (10.0%)
**11–15 yr**	112 (14.7%)	20 (23.8%)	27 (13.5%)	2 (6.9%)	0 (0.0%)
**16–20 yr**	84 (11.0%)	13 (15.5%)	12 (6.0%)	3 (10.3%)	2 (10.0%)
**>20 yr**	394 (51.6%)	33 (39.3%)	126 (63%)	23 (79.3%)	16 (80.0%)

Patients with uncomplicated *P*. *falciparum* malaria were enrolled during the season of high malaria transmission from August to November (2014–2015). The inclusion criteria used during the study were: a) non-complicated malaria patients, b) age: between 3 and 60 years; Parasite counting has been performed in the laboratory of the local dispensary, where patients have been admitted, according to standard procedures. For qPCR analysis 200–300μl of capillary blood were sampled using a finger prick lancet (Accu-Chek, Roche) and collected in an EDTA microtube (Microvette CB300, Sarstedt) and immediately stored at -20°C. Patients were screened and treated on-site daily with standard dihydroartemisinin-piperaquine therapy (Artekin, DP tablets containing 40 mg of dihydroartemisinin and 320 mg of piperaquine phosphate) for three days following the National Malaria Treatment Guidelines [30]. All doses were administered and recorded under the supervision of a qualified member of staff designated by the principal investigator. Patients were supervised and their health conditions were evaluated by continued careful observation. They were screened before the drug treatment on day 0 and after the drug treatment on days 3 and 28 using microscopy and quantitative real-time polymerase chain reaction (qPCR) to evaluate parasitaemia. Drugs were administered to the patients at the local dispensary and patients were asked to return to the dispensary in case of recrudescence. Clinical and laboratory testing were also conducted during the follow-up. To evaluate the rate of asymptomatic carriers in the studied districts during the season of low malaria transmission, blood sampling was conducted from the general population living in the same villages as those of enrolled patients. In Krong Pa, 200 subjects were screened from June to July 2015, while in Huong Hoa, a total of 763 subjects were screened (663 in December 2013 and 100 in December 2015). Malaria recrudescence was retrospectively assessed in malaria patients admitted in the three districts from 2014 to 2016. All patients complied with the follow up study as they were resident in the villages close to the local dispensary.

### National data on malaria collection

The annual data of incidence and prevalence of malaria were collected from:

- The General Statistic Yearbook (Viet Nam) of the years from 2012 to 2016;
- The Annual Reports of the Institute for Malariology, Parasitology and Entomology (Viet Nam) from 2012 to 2016.
- World Health Organization (WHO). National Malaria Programme Review–Viet Nam. 2018

### Laboratory procedures

#### Microscopic analysis of blood smears

Thick blood smears were examined on days 0, 3, and 28 and stained with 10% Giemsa. Parasitaemia was estimated by counting the number of asexual parasites in 500 leucocytes (white blood cells, WBCs), and parasite density (parasites/μL of blood) was then calculated following the guidelines of the WHO, assuming a WBC count of 8,000. Blood smears with discordant results (differences in assessment of species, parasite density >50%, or the presence of parasites) were re-examined by a third independent microscopist, and parasite densities were calculated by averaging the two closest counts [31].

#### *P*. *falciparum* DNA extraction and quantification by qPCR

qPCR was performed on days 0, 3, and 28 on all samples. DNA was extracted from 100 μL of whole blood preserved at -20°C using the salting-out method according to standard procedures [32]. Briefly, red blood cells were lysed with Red Cells Lysis Buffer solution (10 mM Tris-HCl, 5 mM MgCl_2_, and 10 mM NaCl, pH 7.6). Then, the cell pellet was incubated at 55°C for 20 min with White Cells Lysis Buffer solution (Tris-HCl 10 mM, 10 mM ethylenediaminetetraacetic acid, and 50 mM NaCl, pH 7.6) in the presence of 10% sodium dodecyl sulphate and 20 mg/mL proteinase K solution. DNA purification and precipitation were consecutively performed using a saturated salt solution (6M NaCl) and isopropanol, respectively. After washing with 70% ethanol, the DNA pellet was re-suspended in sterile H_2_O. DNA amplification was performed using primers and probes for the 18S rRNA gene of *P*. *falciparum* as previously described with minor modifications [33] DNA was amplified with 1× final reaction buffer, 1.5 mM MgCl_2_, 0.2 mM deoxynucleotide triphosphates (dNTPs), each primer at 300 nM, 100 nM TaqMan probe, and 1 U of Platinum Taq polymerase (Life Technologies, CA, USA).

Amplification and detection were performed using the CFX96 Touch Real-Time PCR Detection System (Biorad) and programmed as follows: 95°C for 3 min, followed by 45 cycles at 95°C for 15 s and 60°C for 45 s. Parasite density (parasites/μL) was estimated using a standard curve obtained from 10-fold serial dilutions of *P*. *falciparum* DNA in culture with known parasitaemia (from 100,000 to 10 parasites/μL), using CFX Manager Software 3.1 (Biorad).

#### Mutations in the K13 propeller domain gene

The *P*. *falciparum* K13 propeller domain gene was amplified by nested PCR as previously described [14]. Amplified products of PCR were purified using Exo I & Fast AP (Carlo Erba), and sequencing was performed by Macrogen (Netherland). Electropherograms were visualised and analyzed with ApoE software and alignments were performed by Muscle 3.8 software with the K13 sequence of the 3D7 clone (PF3D7_1343700) as the reference.

#### *P*. *falciparum* plasmepsin 2 (PfPM2) copy number determination

The copy number of the PfPM2 gene was determined by qPCR as previously described [34], using β-tubulin as an internal standard. The PfPM2 copy number was calculated by the 2-ΔCt method.

#### Molecular diagnosis of red cell mutations

Haemoglobin E (Hb E), haemoglobin constant spring (Hb CS), and glucose-6-phosphate-dehydrogenase (G6PD) deficiency variant Viangchan (G6PD D.) as one of the most common variants throughout South-east Asian populations [35,36] were detected by qPCR using a Taqman probe specific for the wild-type (WT) sequence and one specific for each mutant (MUT) sequence. Primers (forward and reverse) and Taqman fluorescence-labelled probes (WT and MUT) for qPCR were designed using Beacon Designer.

Hb E detection (Ref. Seq: NG_000007.3) was performed using a mixture of 1 mM dNTPs, 3 mM MgCl_2_, each primer at 400 nM (forward: 5′-CCTGAGGAGAAGTCT-3′ and reverse: 5′-TGTCTTGTAACCTTGAT-3′), 125 nM WT probe (5′-[FAM]AGGGCCTCACCAC[BHQ1]-3′), 150 nM MUT probe (5′-[HEX]AGGGCCTTACC[BHQ1]-3′), and 1 U of Platinum Taq DNA (Invitrogen). Amplification was performed under the following conditions: 95°C for 3 min, then 45 cycles at 95°C for 15 s, 60°C for 20 s, and 72°C for 20 s.

G6PD D. detection (Ref. Seq: NG_009015.1) was performed using 1 mM dNTPs, 4 mM MgCl_2_, each primer at 400 nM (forward: 5′-CCAGGACCACATTGTTG-3′ and reverse: 5′-ACCCAAGGAGCCCATTCT-3′),175 nM WT probe (5′-[HEX]ATTTCAACACCTTGACCTGA [BHQ1]-3′), 275 nM MUT probe (5′-[Cy5]ATTTCAACATCTTGACCTGA[BHQ2]-3′), and 1 U of Platinum Taq DNA (Invitrogen). Amplification was performed under the following conditions: 95°C for 3 min, then 45 cycles at 95°C for 15 s, 57°C for 20 s, and 72°C for 20 s.

Hb CS detection **(**Ref. Seq: NG_000006.1) was performed using 10 μL of 2× Taqman (Applied Biosystem) universal master mix, each primer at 400 nM (forward: 5′-GACAAGTTCCTGGCTTCT -3′ and reverse: 5′-ATAGAGAGAACCCAGGCA -3′), 300 nM WT probe (5′-[FAM]GGCTCCAGCTTAACGGTATTTGG[BHQ1]- 3′), and 100 nM MUT probe (5′-[Tx Red]GGCTCCAGCTTGACGGTATTTGG[BHQ1]- 3′) (Invitrogen). Amplification was performed under the following conditions: 50°C for 2 min, 95°C for 10 min, then 45 cycles at 95°C for 15 s, 57°C for 30 s, and 60°C for 30 s. For each protocol, detection and analysis were performed using CFX96 Touch Real-Time PCR Detection System.

#### Statistical analysis

Comparison between categorical variables was performed using a Pearson's chi-square or Fisher exact test, where appropriate (STATA software version 13). Comparison between quantitative variables was performed using Student’s *t*-test (Graph Pad Prism 7.3). A two-tailed p-value of less than 0.05 was considered statistically significant.

## Results

### Variation of malaria cases in three Vietnamese districts

**Fig 1:**
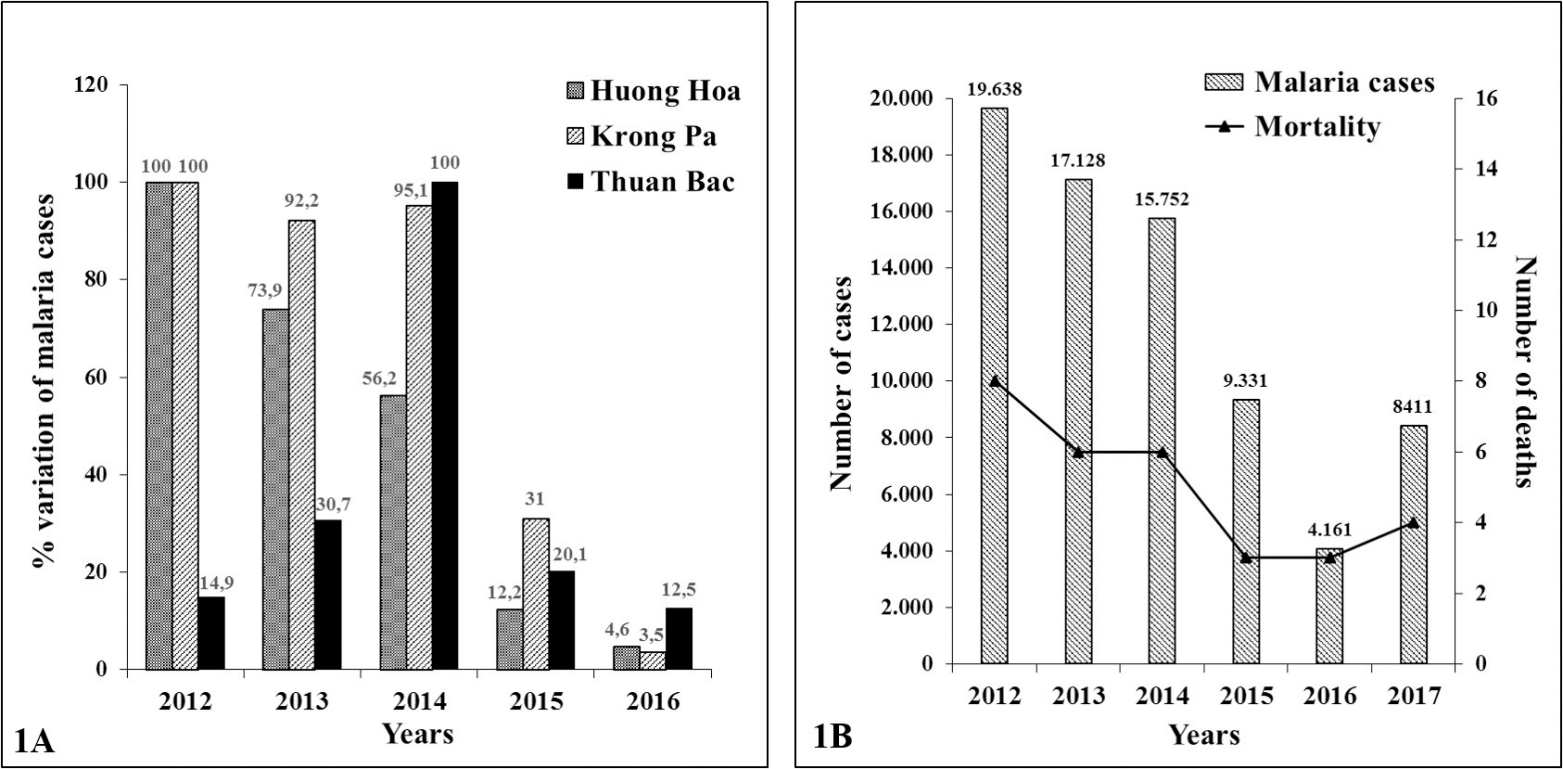
Percentage of variation in P.*falciparum* malaria cases in Huong Hoa, Krong Pa and Thuan Bac districts, from 2012 to 2016 (A). Number of malaria cases and deaths in Vietnam from 2012 to 2017 (B).

Fig 1A shows the percentage variation in the number of *P*. *falciparum* malaria cases from 2012 to 2016 in the three studied districts. Those data are in agreement with the national data (Fig 1B). In Krong Pa and Huong Hoa, the number of malaria cases decreased by more than 95% from 2012 to 2016. In Thuan Bac an abrupt decrease in the number of malaria cases (87.5%) was also observed in the last 2 years which followed an increase in the number of cases from 2012 to 2014. The reason for this biphasic behavior may be influenced by the large number of, immunologically naive, workers that moved in the area from 2012 to 2014.Those data indicate that, in the studied areas, the decline of malaria cases reflects the national data and that control strategies were similarly efficient.

### Prevalence of ART resistance markers in three Vietnamese districts

To verify the clinical impact of the two ART resistance markers, K13 gene mutations and DPC3, we determined their prevalence in three separated districts (Huong Hoa, Krong Pa, Thuan Bac). To exclude the interference that may be exerted by piperaquine resistance, we measured the presence of a specific and sensitive marker of piperaquine resistance (multicopies of PfPM2 gene). In the three districts, DPC3 and K13 mutation rates were measured in a group of *P*.*falciparum* malaria patients. Parasitemia was measured at admission and then on days 3 and 28 by microscopy and qPCR. As expected, microscopy revealed lower sensitivity and accuracy at low parassitemia [37]. The qPCR method was carefully calibrated displaying a limit of detection (LOD) of 1–10 parasites/μl with a coefficient of variation among replicates lower than 30% measured at 100 parasites/μl. qPCR was performed using highly purified DNA extracted from -20°C preserved blood samples rather than blood dried on filter paper because of better sensitivity and reproducibility. It should be noticed that measuring parasitemia by microscopy the rate of DPC3 was approximately 25% lower than by qPCR (Table 2). The mutations on the P.f*alciparum* K13 gene were assessed by DNA sequencing. In accordance with previous reports we distinctively detected the C580Y mutation, the most common mutation on K13 gene found in Vietnam [28].

**Table 2:** Clinical and parasitological parameters (measured by microscopy and qPCR) in Huong Hoa, Krong Pa and Thuan Bac districts.

Parameters	Huong Hoa (n = 84)	Krong Pa (n = 29)	Thuan Bac (n = 20)
**Frequency of positivity on day 3 measured by microscopy and qPCR**	27.2% 39.3%^(*)^	68.1% 86.2%^(^^1^^,^*^)^	32.5% 40.0%^(^*^)^
**Frequency of positivity on day 28 measured by microscopy and qPCR**	0.0% 0.0%^(*)^	8.0% 17.2%^(^^2^^,^*^)^	12.6% 20.0%^(^^2^^,^*^)^
**Frequency of K13 (C580Y)**	1.2%	75.8%^(^^1^^)^	15.0%
**Frequency of PfPM2 multi-copies**	1.2%	0.0%	0.0%
**Mean parasitemia (parasites/μl) on day 0 by microscopy and qPCR**	25423 33839(*)	59223 73801(*)	33665 45997(*)
**Mean parasitemia (parasites/μl) in positive patients on day 3 by qPCR**	100	165	81
**Mean parasitemia (parasites/μl) in positive patients on day 28 by qPCR**	—	18	14
**Frequency of malaria recrudescence on day 60**	0.0%	0.0%	0.0%
**Percent decrease of the number of malaria cases in the last years**	95.0% (from 2012)	97.0% (from 2012)	87.0% (from 2014)
**Frequency of asymptomatic carriers**	1.2%	1.0%	—

*n*: number of studied patients.

^(1)^ The value is significantly different (p < 0.05) in comparison to the two other values belonging to the same category.

^(2)^ The value is significantly different (p < 0.05) in comparison to the lowest value belonging to the same category. The frequency of malaria recrudescence was retrospectively assessed in malaria patients admitted in the three districts from 2014 to 2016.

^(*)^ Frequency of positivity on days 3 and 28 measured by qPCR.

Table 2 shows the largely different rates of ART resistance markers measured in the three districts by microscopy and qPCR. The mean parasitemia levels at admission and on day 3 were higher in Krong Pa than in the other two districts. By qPCR on day 28, we observed the presence of circulating parasites only in Krong Pa and in Thuan Bac. At microscopic examination we could not evidence the presence of gametocytes but due to extremely low parasite density observed by qPCR we cannot exclude their eventual presence. Table 2 shows nearly absence of piperaquine resistance in all studied districts. Resistance has been searched using a sensitive and specific molecular marker (multi-copies of PfPM2 gene) (S1 Fig); (S1 Row Data).

#### Evaluation of the impact of ART resistance on treatment effectiveness

According to a WHO classification [12] we divided the patients in 3 groups: A) no ART resistance (absence of both AR resistance markers), B) suspected endemic resistance (presence of only one marker), C) confirmed endemic resistance to ART (presence of both markers) (Table 3). Independently to the different degrees of confirmed ART resistance, in the three districts we did not evidence signs of clinical treatment failure both in the enrolled patients and in the three districts evaluating the official medical records for recrudescence within 60 days after treatment (from 2014 to 2016) (Table 2). Although unlikely, as many patients live in a village close to a medical dispensary where antimalarial drugs are administered, self-medication cannot be totally excluded. Table 3 also shows that group C displays much higher parasitemia at admission than groups A and B accompanied by higher rates of patients with circulating parasites on day 28.

**Table 3:** Clinical and parasitological parameters in the patients grouped according to WHO definition of endemic ART resistance (*n*: Number of patients).

	No ART Resistance	(B) Suspected endemic ART Resistance	(C) Confirmed endemic ART resistance
Parameters	*n* = 63	*n* = 48	*n* = 22
**Mean parasitemia** **(parasites/**μ**l) on day 0**	26004	47703	89759
**Mean parasitemia (parasites/μl) in positive patients on day 3**	0	95	176
**Mean parasitemia(parasites/μl) in positive patients on day 28**	0	14	18
**Frequency of DPC on day 28**	0%	8.3%	22.7%

### Effect of potential confounding factors on the assessment of DPC3

We analyzed the interference of some potential confounding factors on the rate of clearance of parasites. The prevalence of some common human mutations selected by malaria such as G6PD deficiency variant Viangchan, Haemoglobin E and Haemoglobin Constant Spring were measured in the general population and in malaria patients. Table 4 shows that the prevalence of the studied mutations was not significantly different in the general population and in malaria patients which displayed DPC3. This observation indicates that those mutations do not apparently affect the parasite clearance rate (p > 0.05). It should be, anyway noticed that a larger cohort of patients should be studied to increase the statistical power of the results. The mean age of the patients displaying DPC3 was similar to the age of patients without DPC3 (23.7 vs 25.9; p > 0.05). On the contrary, a subset of patients characterized by a slower clearance of parasites on day 3 (more than 5% of infected parasites still present on day 3) showed a significantly lower (p < 0.05) mean age (14.0 years) than patients presenting faster parasite clearance (24.7 years). The gender of patients did not play a role on DPC3. In conclusion we could not identify any factor that may clearly affect the different distribution of ART resistance markers observed in the studied districts. In addition, also the effectiveness of malaria control measures such as: bed nets, vector control, and quality of health system, appear homogeneous in the three districts limiting their possible interference.

**Table 4:** Comparison of frequency (%) of human mutations (G6PD Deficiency, Haemoglobin E, and Haemoglobin Constant Spring) between the general population and malaria patients with and without DPC (*n* = number of patients).

	G6PD Deficiency variant Viangchan (%)	Haemoglobin E (%)	Haemoglobin Constant spring (%)
**General population** **(*n* = 400)**	9.5	51.5	28.7
**Malaria patients** **with DPC3** **(*n* = 66)**	10.6	45.4	28.7
**Malaria patients** **without DPC3** **(*n* = 67)**	7.5	59.7	34.3

## Discussion

In the past years a sustained decline in the number of *P*. *falciparum* malaria cases has been observed in Vietnam. On the other hand, the increasing rates of ART resistance markers, the appearance of piperaquine resistance at the Cambodian border [23,38], the threat of multidrug resistant parasites and a moderate increase of malaria cases observed in 2017 in Vietnam, are raising concern. On the other hand, the actual relevance of the ART resistance markers in predicting the risk of treatment failure is still debated [15], the significance of artemisinin resistance is constantly updated [12,39–46] and the available literature is sometime incomplete or contradictory[8,11,16,20–25]. To establish the impact of ART resistance on clinical treatment failure we followed a comprehensive, in dept, approach: we measured the rates of suspected endemic artemisinin resistance and confirmed endemic artemisinin resistance according to the current WHO guidelines[12,45] to identify areas with markedly different prevalence of confirmed ART resistance (from 1.7% to 75.8%). In the studied districts we assessed the prevalence of clinical treatment failure (recrudescence within 60 days) in a large number of subjects. We also tested PPQ resistance by a validated marker in all districts. Some frequent human mutations known to affect parasite clearance and the efficacy of malaria control strategies were evaluated in order to exclude some major confounding factors. In the present report parasitemia was measured by microscopy to allow easier comparison with previous reports and by qPCR to allow the quantification of parasites on days 3 and 28. In addition, qPCR revealed that K13 (C580Y) variant is associated to much higher parasitemia at admission. In all studied districts we could not evidence clinical treatment failure (no cases of clinical recrudescence within two months from the treatment). Our data tend to exclude interferences caused by human mutations affecting erythrocyte functions or differences of malaria control effectiveness in the studied districts.

In the two districts displaying the highest and the lowest prevalence of the K13 mutation, we observed markedly different rates of DPC3 (86.2% vs 39.3%) confirming the association between the two markers [8,11,20,24]. In patients with parasites mutated at K13 (C580Y), persistence of parasites on day 28 was observed. Interestingly, by quantitative parasite measurement we evidenced that K13 (C580Y) mutation is strongly associated to higher parasite density at admission, on day 3 and to the presence of residual parasites on day 28 (S1 Fig). The lack of clinical treatment failure in presence of DPC may be due to the very low parasite density of residual parasites (from 81 to 165 parasites/ul on day 3 and from 14 to 18 parasites/μl on day 28). It should be anyway noticed that we cannot exclude that residual parasites were not gametocytes, moreover we cannot provide evidence on the viability of the parasites at such low densities It should be also taken in consideration that piperaquine reaches its peak plasma concentration on day 3–4 and possess a very prolonged half-life (four weeks). Piperaquine may, therefore, kill the very few and, plausibly, damaged residual parasites observed on days 3 and 28.

The use of quantitative measurements provided additional information: i) the co-occurrence of both ART resistance markers was associated to a 3.4 fold higher parasite density at admission in comparison to patients displaying no ART resistance markers; ii) patients displaying a slower clearance of parasites on day 3 (< 95% of initial density) were significantly younger than patients displaying faster clearance. Those observations are in accordance with reports indicating that immunity is a major determinant of the parasite clearance rate [47–49] that, in turn, is expected to influence parasitemia. As expected, a reduction of malaria immunity has been described [48] in large regions of the GMS, including Vietnam, where malaria transmission substantially declined in the past 5–10 years. Interestingly, the same report provides strong evidence that K13 (C580Y) mutation is associated to decreased immunization, suggesting that lack of acquired immunity may affect the selection on K13 mutations and cause delayed parasite clearance. It should be also noticed that DPC may promote the spread of parasites resistant to the partner drug [49] and that resistance to the partner drugs clearly associated to ACT resistance [50].

In conclusion ART resistance markers are not predictive of clinical failure [24,25]. This result was not obvious because the available literature is still incomplete or provides conflicting results[8,11,16,20–25] in addition, the last WHO reports don’t exclude treatment failure in presence of ART resistance[12,45,46]. Anyway, independently on the causes, more effective treatments are required to counteract the phenomenon of delayed parasite clearance.

## Supporting information

S1 Fig Plasmepsin2. (PDF)

S1 Row Data Row Data K13 Sequences. (RAR)
