# Supplementary material for: Clinical impact of the two ART resistance markers, K13 gene mutations and DPC3 in Vietnam
Source: PLoS One. 2019 Apr 2;14(4):e0214667. doi: 10.1371/journal.pone.0214667 (PMC6445423; doi:10.1371/journal.pone.0214667)
Supplement: S1 Fig — (PDF) [file pone.0214667.s001.pdf]

pasmepsin2 amplification HUONG HOA PATIENT.pcrd

07/02/2019 15:04

Report Information

User: BioRad/admin
Data File Name: pasmepsin2 amplification HUONG HOA PATIENT.pcrd
Data File Path: C:\Users\Maria Carmina Pau\Desktop\PAPER 19 ALLEGATI\supplementary data
Well Group Name: Group 1
Report Differs from Last Save: No

Run Setup

Run Information

Run Date: 06/05/2017 21:59
Run User:
Run Type: User-defined
Plate File: DefaultPlate.pltd
ID:
Notes:
Sample Volume: 20
Temperature Control Mode: Calculated
Lid Temperature: 105
Base Serial Number: CT004529
Optical Head Serial Number: 785BR8775

Protocol

1: 50,0°C for 2:00
2: 95,0°C for 10:00
3: 95,0°C for 0:15
4: 60,0°C for 0:30
5: 60,0°C for 0:30
Plate Read
6: GOTO 3, 40 more times

Plate Display

	1	2	3	4	5	6	7	8	9	10	11	12
A	Unk<br>PM2<br>NT001	Unk<br>B TUB<br>NT001	Unk<br>PM2<br>VAS03A	Unk<br>B TUB<br>VAS03A	Unk<br>PM2<br>VCQ 03	Unk<br>B TUB<br>VCQ 03	Unk<br>PM2<br>X29	Unk<br>B TUB<br>X29				
B	Unk<br>PM2<br>NT 002	Unk<br>B TUB<br>NT 002	Unk<br>PM2<br>VCQ05	Unk<br>B TUB<br>VCQ05	Unk<br>PM2<br>VCQ02	Unk<br>B TUB<br>VCQ02	Unk<br>PM2<br>T25	Unk<br>B TUB<br>T25				
C	Unk<br>PM2<br>NT 004	Unk<br>B TUB<br>NT 004	Unk<br>PM2<br>VCQ06	Unk<br>B TUB<br>VCQ06	Unk<br>PM2<br>VAL04	Unk<br>B TUB<br>VAL04	Unk<br>PM2<br>X43	Unk<br>B TUB<br>X43				
D	Unk<br>PM2<br>NT007	Unk<br>B TUB<br>NT007	Unk<br>PM2<br>VCQ07	Unk<br>B TUB<br>VCQ07	Unk<br>PM2<br>VCQ03H	Unk<br>B TUB<br>VCQ03H	Unk<br>B TUB<br>huong hoa<br>30	Unk<br>PM2<br>huong hoa<br>30				

Plate Display

	1	2	3	4	5	6	7	8	9	10	11	12
E	Unk<br>PM2<br>NT08	Unk<br>B TUB<br>NT08	Unk<br>PM2<br>VAS04	Unk<br>B TUB<br>VAS04	Unk<br>PM2<br>VCQ08	Unk<br>B TUB<br>VCQ08	Unk<br>PM2<br>X18	Unk<br>B TUB<br>X18				
F	Unk<br>PM2<br>NT 10	Unk<br>B TUB<br>NT 10	Unk<br>PM2<br>VAL3C	Unk<br>B TUB<br>VAL3C	Unk<br>PM2<br>VCQ07	Unk<br>B TUB<br>VCQ07	Unk<br>PM2<br>X28	Unk<br>B TUB<br>X28				
G	Unk<br>PM2<br>VCQ 04	Unk<br>B TUB<br>VCQ 04	Unk<br>PM2<br>VAS05	Unk<br>B TUB<br>VAS05	Unk<br>PM2<br>X44	Unk<br>B TUB<br>X44	Unk<br>PM2<br>X4	Unk<br>B TUB<br>X4				
H	Unk<br>PM2<br>B10	Unk<br>B TUB<br>B10	Unk<br>PM2<br>B13	Unk<br>B TUB<br>B13	Unk<br>PM2<br>BT20	Unk<br>B TUB<br>BT20	Unk<br>PM2<br>B12	Unk<br>B TUB<br>B12				

Quantification

Step #: 5
Analysis Mode: Fluorophore
Cq Determination: Single Threshold
Baseline Method:
SYBR: Auto Calculated
Threshold Setting:
SYBR: 310,60, User Defined

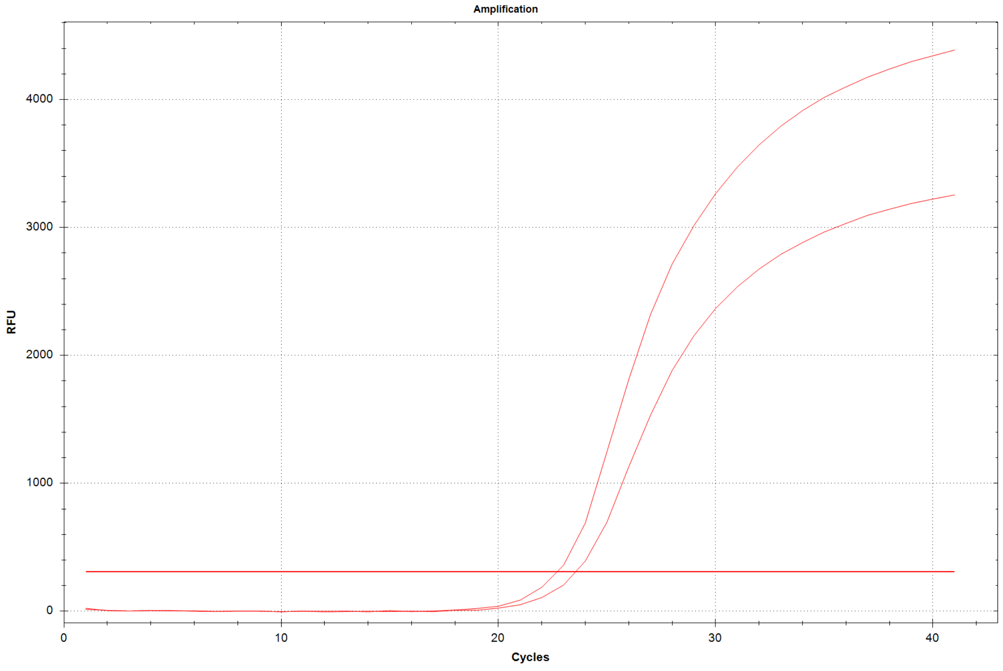

Quantification Data

Well	Fluor	Target	Content	Sample	Cq	Cq Mean	Cq Std. Dev
D07	SYBR	B TUB	Unkn	huong hoa 30	23,56	23,56	0,000
D08	SYBR	PM2	Unkn	huong hoa 30	22,71	22,71	0,000

Bar Chart

Expression analysis is not possible, wells must contain at least two samples with target(s), which have a valid Cq.

Target Names

Name	Full Name	Reference	Auto Efficiency	Efficiency
B TUB	B TUB	False	Yes	100,0%
PM2	PM2	False	Yes	100,0%

Sample Names

Name	Full Name	Control
huong hoa 30	huong hoa 30	Yes

Gene Expression - Bar Chart Data

Target	Sample	Ctrl	Relative Quantity	Relative Quantity SEM	Corrected Relative Quantity SEM	Mean Cq	Cq SEM
B TUB	huong hoa 30		0,00000	N/A	N/A	23,56	0,00000
PM2	huong hoa 30		0,00000	N/A	N/A	22,71	0,00000

QC Parameters

Data

Description	Value	Use	Results	Exclude Wells	All excluded wells
Negative control with a Cq less than	38	True		False	
NTC with a Cq less than	38	True		False	
NRT with a Cq less than	38	True		False	
Positive control with a Cq greater than	30	True		False	
Unknown without a Cq	N/A	True		False	
Standard without a Cq	N/A	True		False	
Efficiency greater than	110,0	True			
Efficiency less than	90,0	True			
Std Curve R^2 less than	0,980	True			

Data

**Description**	**Value**	**Use**	**Results**	**Exclude Wells**	**All excluded wells**
Replicate group Cq Std Dev greater than	0,20	True		False	
